# Identification of diagnostic markers pyrodeath-related genes in non-alcoholic fatty liver disease based on machine learning and experiment validation

**DOI:** 10.1038/s41598-024-77409-3

**Published:** 2024-10-26

**Authors:** Liping Lei, Jixue Li, Zirui Liu, Dongdong Zhang, Zihan Liu, Qing Wang, Yi Gao, Biwen Mo, Jiangfa Li

**Affiliations:** 1 Department of Geriatric Medicine, The Affiliated Hospital of Guilin Medical University, Guilin, 541001 Guangxi China; 2 Division of Hepatobiliary Surgery, The Affiliated Hospital of Guilin Medical University, Guilin, 541001 Guangxi China; 3 Department of Gastrointestinal Surgery, The Affiliated Hospital of Guilin Medical University, Guilin, 541001 Guangxi China; 4 Department of Respiratory and Critical Care Medicine, The Second Affiliated Hospital of Guilin Medical University, Guilin, 541002 Guangxi China; 5 Key Laboratory of Early Prevention and Treatment for Regional High Frequency Tumor, Guangxi Medical University, Ministry of Education, Nanning, 530021 Guangxi China; 6 Guangxi Key Laboratory of Early Prevention and Treatment for Regional High Frequency Tumor, Nanning, 530021 Guangxi China

**Keywords:** Non-alcoholic fatty liver disease, Pyroptosis, Diagnostic, Immune infiltration, Machine learning, Genomics, Computational biology and bioinformatics, Biomarkers, Gastroenterology

## Abstract

Non-alcoholic fatty liver disease (NAFLD) poses a global health challenge. While pyroptosis is implicated in various diseases, its specific involvement in NAFLD remains unclear. Thus, our study aims to elucidate the role and mechanisms of pyroptosis in NAFLD. Utilizing data from the Gene Expression Omnibus (GEO) database, we analyzed the expression levels of pyroptosis-related genes (PRGs) in NAFLD and normal tissues using the R data package. We investigated protein interactions, correlations, and functional enrichment of these genes. Key genes were identified employing multiple machine learning techniques. Immunoinfiltration analyses were conducted to discern differences in immune cell populations between NAFLD patients and controls. Key gene expression was validated using a cell model. Analysis of GEO datasets, comprising 206 NAFLD samples and 10 controls, revealed two key PRGs (TIRAP, and GSDMD). Combining these genes yielded an area under the curve (AUC) of 0.996 for diagnosing NAFLD. In an external dataset, the AUC for the two key genes was 0.825. Nomogram, decision curve, and calibration curve analyses further validated their diagnostic efficacy. These genes were implicated in multiple pathways associated with NAFLD progression. Immunoinfiltration analysis showed significantly lower numbers of various immune cell types in NAFLD patient samples compared to controls. Single sample gene set enrichment analysis (ssGSEA) was employed to assess the immune microenvironment. Finally, the expression of the two key genes was validated in cell NAFLD model using qRT-PCR. We developed a prognostic model for NAFLD based on two PRGs, demonstrating robust predictive efficacy. Our findings enhance the understanding of pyroptosis in NAFLD and suggest potential avenues for therapeutic exploration.

**Supplementary Information:**

The online version contains supplementary material available at 10.1038/s41598-024-77409-3.

## Introduction

Non-alcoholic fatty liver disease (NAFLD), representing the most prevalent liver condition, emerges as a significant global health dilemma, implicating up to a quarter of the adult populace worldwide. This disease not only underscores the escalating burden on healthcare infrastructures globally but also highlights an alarming trend of increased incidences among the pediatric demographic^1^. NAFLD is defined by the deposition of fat exceeding 5% in liver cells, in scenarios devoid of substantial alcohol intake or other secondary hepatic steatosis triggers such as obesity, dyslipidemia, type 2 diabetes, and assorted metabolic disorders^2^. NAFLD embodies a spectrum of hepatic abnormalities ranging from mere fat aggregation (benign steatosis) to more severe forms including inflammation (Non-alcoholic steatohepatitis [NASH]), fibrosis, cirrhosis, and ultimately, hepatocellular carcinoma^3,4^. In the context of immune response, inflammation fundamentally serves as a protective measure against external pathogens^5^. Nonetheless, it has been demonstrated through prior research that inflammation, particularly when provoked by immune cytokines, can inflict significant tissue damage^6^.

Pyroptosis is a programmed cell death induced by a typical or atypical inflammasome, which is morphologically manifested as cell swelling and subsequent lysis, ultimately resulting in the release of intracellular contents^7,8^. Pyroptosis is regulated by unique sets of critical inflammatory caspases that coordinate biological effects^9,10^. Inflammasomes are multiprotein complexes that can sense danger signals and activate caspase-1 to mediate pro-inflammatory cytokines release and pyroptotic cell death. There are two main canonical and non-canonical signaling pathways that trigger inflammasome activation^11^. Pyroptosis is involved in many pathophysiological processes^8,12,13^. Pyroptosis is involved in liver fibrogenesis from various pathologies^14,15^.

There is a relative scarcity of research delving into the connection between pyroptosis metabolism and the underlying pathophysiology of NAFLD. To address this, we extensively investigated the expression, diagnosis, immune correlation, and mechanism of pyroptosis-related genes (PRGs) in NAFLD. Utilizing NAFLD-related data downloaded from the Gene Expression Omnibus (GEO) database, we conducted a series of analyses, including machine learning, to elucidate the relationship between PRGs and NAFLD. This process allowed us to identify differentially expressed genes, pinpoint key genes among them, and construct a prediction model that was subsequently externally validated. In the final stages of our study, we carried out immune infiltration analyses, evaluated related drugs, and explored associated competing endogenous RNAs (ceRNAs).

## Materials and methods

### Patients and datasets

**Fig. 1:**
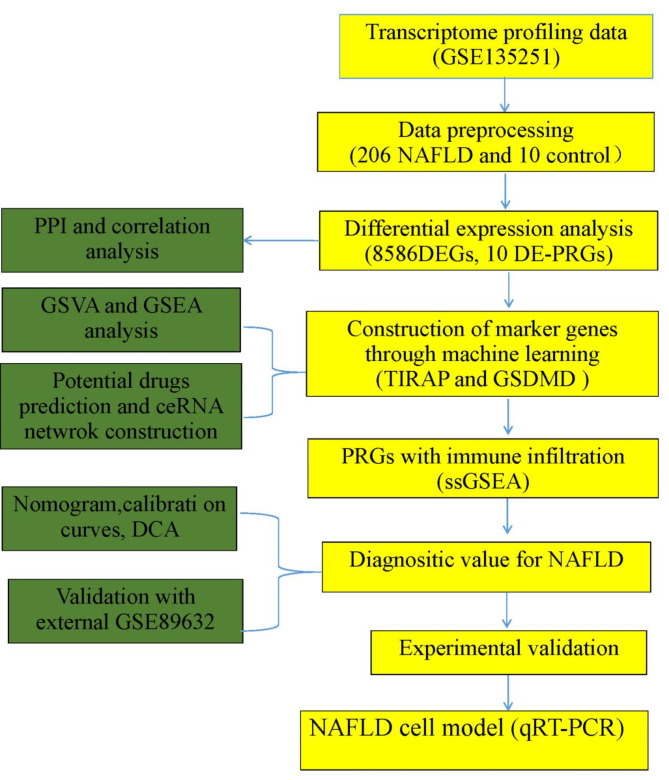
Flowchart of the present study.

The transcriptomic analysis of NAFLD and normal liver specimens included GSE135251, downloaded from the GEO database (53steatosis, 153 NASH and 10 control samples). The flow chart of this study is shown in Fig. 1. The identification of effective genes took the following steps. Firstly, genes with missing values were deleted. Secondly, genes with multiple duplicate values were averaged. Furthermore, if a gene had a value of 0 in half or more of the samples, the gene was deleted. The remaining valid genes were then used for differential expression analysis.

### Expression of DEGs and PRGs in NAFLD

We utilized the R package “limma” to conduct a differential expression analysis based on the processed data from the GEO database. This analysis allowed us to identify differentially expressed genes (DEGs) between NAFLD samples and healthy controls. The results were visualized as volcano and heatmap plot using the “ggplot2” and “heatmap” R packages. The screening criteria were as follows: adjusted *P* < 0.05. The intersection of DEGs related to PRGs was created using the “VennDiagram” R package and defined as DEG-PRGs for subsequent analysis. The Ven map was used to identify PRGs in DRGs between NAFLD and healthy samples. The “ggpubr” R package was use to build a boxplot representing the differences expressed of the DEG-PRGs between NAFLD and normal groups. The R software packages “heatmap” was used to generate heatmap of the DE-PRGs. Then, we used multivariate analysis to screen for important DE-PRGs.

### Correlation analysis and protein‒protein interaction (PPI) network construction

The DE-PRGs PPI networks were constructed using the Search Tool for the Retrieval of Interacting Genes/Proteins (STRING; https://string-db.org/) database. The “ggplot2[3.3.6]” package of R was used to carry out pair-to-pair correlation analysis of variables in the data, and the analysis results were visualized with the heat map.

### Gene ontology (GO) and KEGG pathway enrichment analysis

To analyze the biological function of genes, we employed the “clusterProfiler” package in R, which facilitated the enrichment analysis of Gene Ontology (GO) and Kyoto Encyclopedia of Genes and Genomes (KEGG) pathways^16–18^. The GO annotation encompassed three domains: biological processes (BP), cellular components (CC), and molecular functions (MF).

### Three machine learning methods to identify key genes

The “glmnet” package in R was employed to perform the least absolute shrinkage and selection operator (LASSO) regression on the selected linear model, a method that reduces data dimensionality while retaining valuable variables^19,20^. The principle of recursive feature elimination (RFE) is to iteratively build the model and then select the best (or worst) feature, determined by the coefficient^21^. The sequential backward selection algorithm, known as support vector machine recursive feature elimination (SVM-RFE), is based on the maximum margin principle of the support vector machine (SVM)^22–24^. SVM-RFE is a supervised machine learning model that differentiates between positive and negative instances by removing the feature vector generated by SVM. SVM is a powerful supervised learning algorithm used for classification and regression tasks. The primary goal of SVM is to find the optimal hyperplane that separates data points of different classes with the maximum margin. In bioinformatics, SVM is used for gene expression data analysis, protein classification, and cancer classification. SVM can extract features from complex biological data for effective classification prediction. RFE systematically removes features to improve the model’s performance, thus helping to identify a subset of genes that are most informative for distinguishing between classes. We used the “e1071” package in R to screen the most valuable genes for the SVM-RFE model construction^25,26^. The SVM-RFE method was utilized to determine the optimal variables by searching for the point corresponding to the minimum cross-validation error. The random forest (RF) algorithms were integrated to select the optimal genes. RF is a regression tree technique that leverages bootstrap aggregation and randomization of predictors to achieve high prediction accuracy^27^. The “randomForest” R package was utilized for RF. The genes selected by these three machine learning methods were intersected to obtain the final key genes.

### The establishment and verification of the DE-PRGs diagnostic model

In the construction of the DE-PRGs diagnostic model, we utilized the GSE135251 dataset for model construction. Initially, we utilized the “rms” package in R to construct a nomogram model to predict the occurrence of NAFLD. The “pROC” package in R^28^ was employed to analyze the area under the curve (AUC), specificity, and sensitivity of diagnostic value for marker genes using a time-dependent ROC. Each central gene was assigned a score, which was then aggregated to form a total score. The external dataset, GSE89632, was used to verify the diagnostic capability of the model. The GSE89632 dataset included 20 cases of simple steatosis, 19 cases of nonalcoholic steatohepatitis, and 24 healthy controls.

### Gene set enrichment analysis (GSEA) and gene set variation analysis (GSVA)

We conducted GSEA using the “clusterProfiler” package to explore the potential functions of the hub genes^29^. Additionally, we executed differential gene expression and pathway enrichment analyses using the “GSVA,” “clusterProfiler,” and “Limma” packages. Statistical significance was determined by enrichment analysis with a p-value less than 0.05.

### Assessment of Immune Infiltration

The “GSVA” package in R was utilized to apply a single-sample GSEA (ssGSEA) algorithm for determining enrichment scores between diverse immune cells, functions, or pathways in NAFLD and control groups. Reference gene collections were obtained from a public database (http://www.immport.org). The association between the four pivotal genes and the immune score was investigated using Spearman correlation analysis. The Wilcoxon test was employed to examine differences in immune cell and immune-related functional enrichment scores.

### Investigation of drug-gene interactions and construction of ceRNA network

The drug-gene interaction database (DGIdb, https://dgidb.genome.wustl.edu/) was used to probe drug-gene interactions^30^. The miRNA-mRNA relationship could be predicted by leveraging the two key genes in miRDB (http://www.mirdb.org/) and TargetScan (https://www.targetscan.org/vert_80). Direct interaction evidence between the miRNA and lncRNA was gathered using SpongeScan (http://spongescan.rc.ufl.edu/). The Cytoscape software (version 3.9.0) was used to visualize mRNA‒miRNA–lncRNA interactions through the ceRNA network.

### Cell culture and treatments

The Human Liver-7702 (HL-7702) cell line was supplied by Cybkon Biotechnology Co., LTD., (Shanghai, China, Item number: iCell-h054). The complete culture medium of HL-7702 cell line was added with DMEM/F-12 (1:1) (Gibco, 11330-032) 89 mL ITS liquid medium (Sigmadg, I3146) 1 mL dexamethasone (Sigma, D4902-100 mg) 40 ng/ mL FBS (Gibco) 10 mL, 37uC, 5% CO2, cultured in a cell incubator. When the cell fusion degree reached 60–70%, the cells were divided into 2 groups (*n* = 3). (1) Control group (NS treatment for 24 h); (2) NAFLD group (cell treated with oleic acid (OA; Sigma, USA) 1mM for 24 h.). Cell condition was confirmed by Oil Red O staining.

### Quantitative reverse transcription-polymerase chain reaction (qRT–PCR)

The RNA was extracted from HL-7702 cell line with TRIzol reagent (VAZYME, China). RNA was extracted and eluted through an RNA binding column, yielding purified total RNA samples. The first strand cDNA synthesis kit was used for cDNA reverse transcription. SYBR green qRT–PCR premix was employed for qRT–PCR. The expression levels of the target genes were normalized and analyzed in relation to GAPDH expression. The PrimeScript™ RT Reagent Kit (VAZYME, China) was used for RNA reverse transcription, and qRT‒PCR was carried out with an FX Connect system(VAZYME, China) and SYBR^®^ Green Supermix (VAZYME, China). qRT‒PCR was performed in triplicate. The primers used in this study are listed in Supplementary Table S1.

### Statistical analyses

Continuous variables are expressed as mean ± standard deviation. The Student’s t-test was used for comparing two groups, while the Wilcoxon rank-sum test was used for analyzing non-normally distributed variables. A p-value less than 0.05 was deemed to indicate a significant difference. The symbols *, **, and *** denote p-values less than 0.05, 0.01, and 0.001, respectively. All statistical analyses were conducted using R software (version 4.2.1).

## Results

### Identification of DE-PRGs associated with NAFLD

**Fig. 2:**
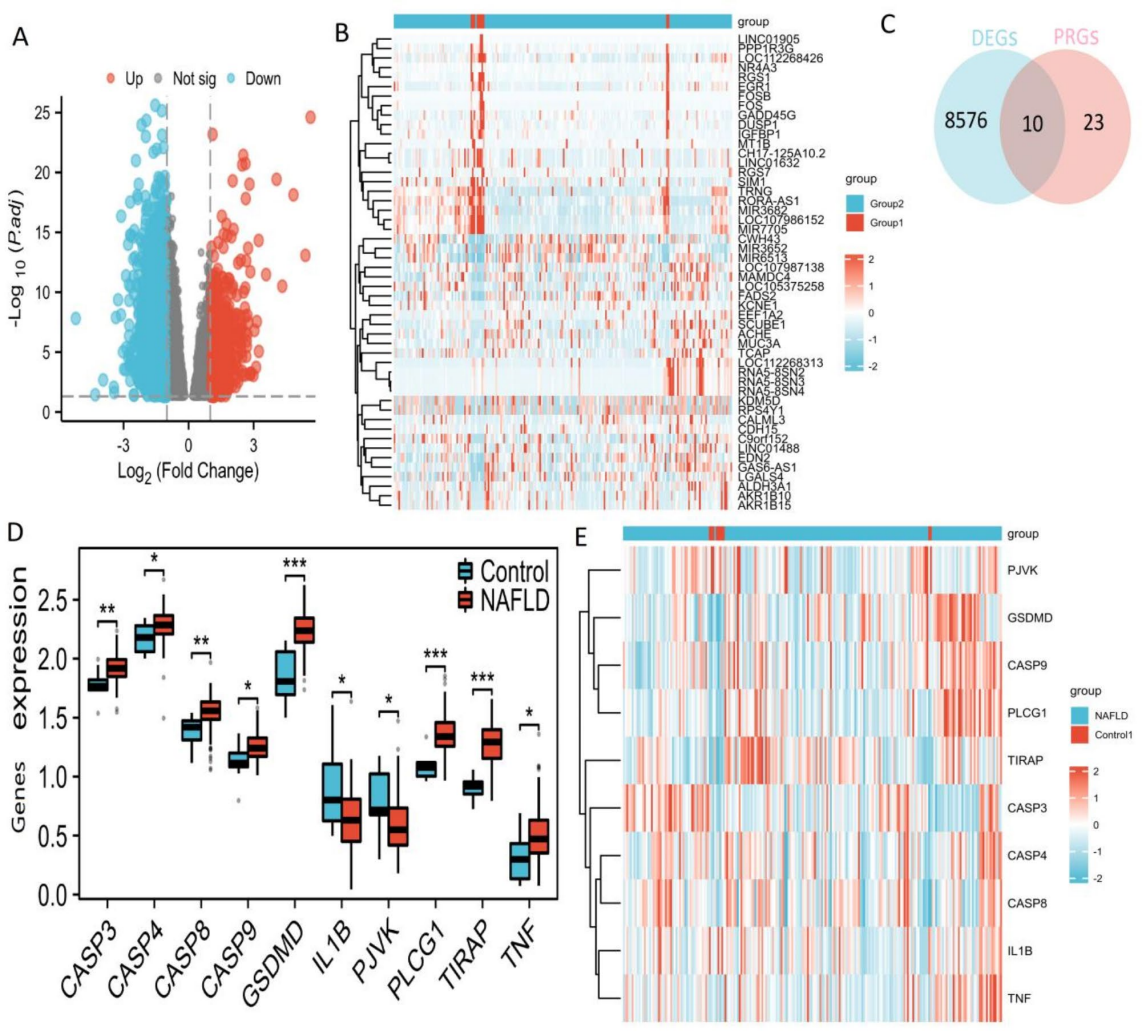
Identification of DE-PRGs in NAFLD. (**A**) Volcano plot of DEGs between NAFLD and control samples. (**B**) Heatmaps of the top 50 genes are presented. The top 50 genes included the top 25 DEGs with the largest values for positive logFC and the top 25 DEGs with the largest absolute values for negative logFC. (**C**) Venn diagrams showing the intersection between DEGs and PRGs. (**D**) Ten DE-PRGs are presented with the boxplots illustrating the differential expression between NAFLD and control samples. (**E**) Heatmap showing the expression patterns of these 10 DE-PRGs. P values are displayed as follows: *p < 0.05; **p < 0.01; ***p < 0.001. *DEGs* differentially expressed genes, *DE-PRGs* differentially expressed pyroptosis-related genes, *PRGs* pyroptosis-related genes, *NAFLD* nonalcoholic fatty liver disease.

Using the “limma” package, 8586 DEGs (adj.*p* < 0.05) were identified from the GSE135251 dataset consisting of 206 NAFLD and 10 control samples, as shown in Supplementary Table S2. Of these, 895 genes were up-regulated, and 1364 were down-regulated (Supplementary Table S3). The volcano plot of the differentially expressed genes is shown in Fig. 2A, and the heatmap of the top 50 genes in NAFLD and control samples is displayed in Fig. 2B. The top 50 genes included the top 25 DEGs with the largest values for positive logFC and the top 25 DEGs with the largest absolute values for negative logFC in DEGs. Additionally, 33 PRGs^31^ overlapped with the 8586 DEGs, revealing 10 DE-PRGs with significant differences between the NAFLD and control groups (Fig. 2C, Supplementary Table S4). Eight DE-PRGs (CASP3, CASP4, CASP8, CASP9, GSDMD, PLCG1, TIRAP, TNF) were high expression and two DE-PRGs (IL1B and PJVK) were low expression in NAFLD (Fig. 2D, Supplementary Table S5), and the heatmap of these 10 DE-PRGs was shown in Fig. 2E.

**Fig. 3:**
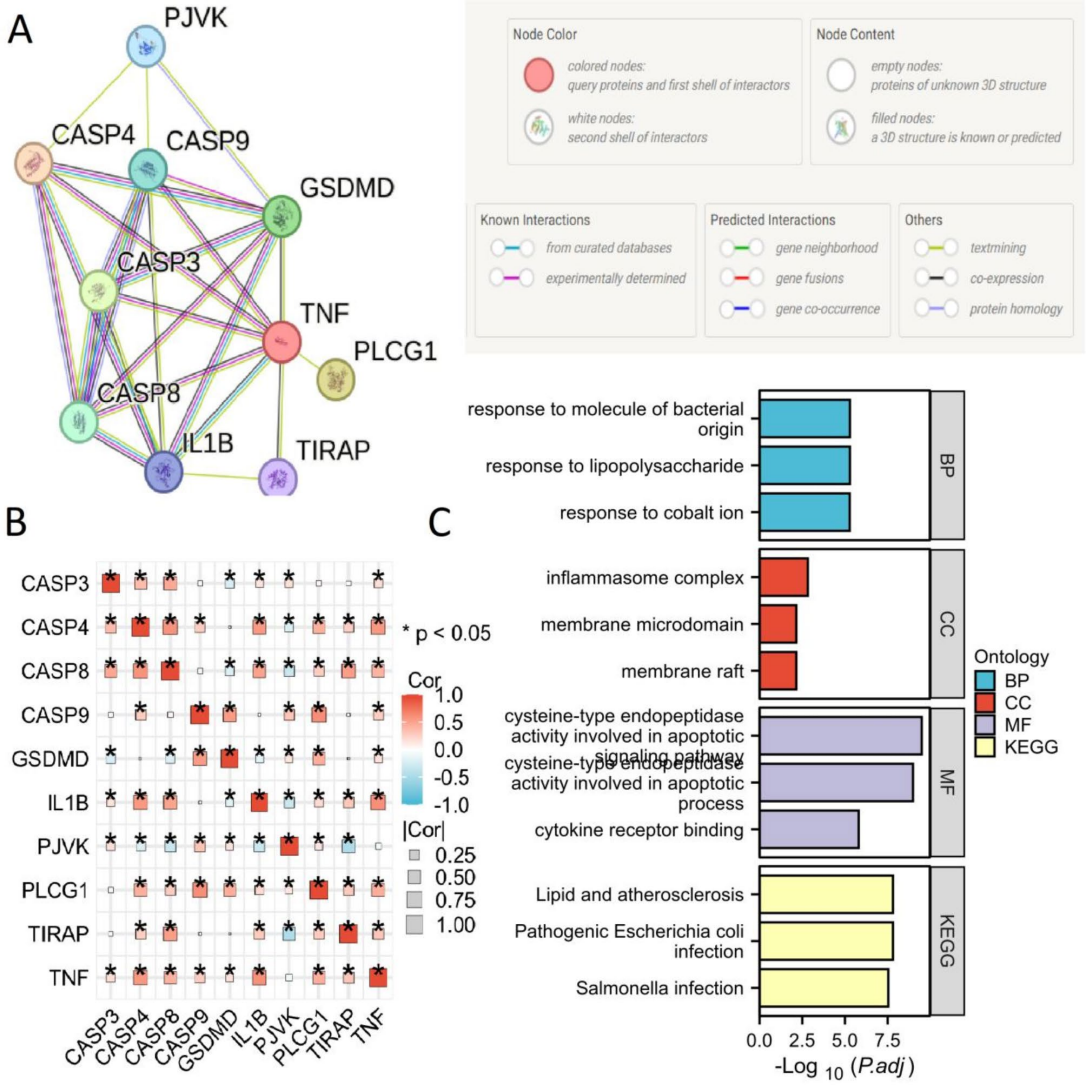
PPI, GO, and KEGG analysis of 10 DE-PRGs. (**A**) Gene relationship network diagram of the 10 DE-PRGs. (**B**) Correlation analysis of the 10 DE-PRGs was conducted, with orange and blue representing positive and negative correlations, respectively. (**C**) GO and KEGG analysis of 10 DE-PRGs. P values are displayed as follows: *p < 0.05. *DEGs* differentially expressed genes, *DE-PRGs*: differentially expressed pyroptosis-related genes, *BP* biological processes, *CC* cellular components, *MF* molecular functions, *GO* gene ontology, *KEGG* Kyoto Encyclopedia of Genes and Genomes.

A PPI analysis using STRING was conducted to explore potential interactions among these 10 DE-PRGs (Fig. 3A). The correlation among the 10 DE-PRGs is shown in Fig. 3B. DE-PRGs were found to be related to response to lipopolysaccharide (LPS), molecule of bacterial origin, cobalt ion, and NF-kappaB signaling in BP, and inflammasome complex, membrane raft and membrane microdomain in CC, and cysteine-type endopeptidase activity, cytokine receptor binding, and cytokine receptor binding in MF, as revealed by GO enrichment analysis (Fig. 3C, Supplementary Table S6). KEGG pathway analysis showed involvement in Pathogenic Escherichia coli infection, lipid and atherosclerosis, liver disease, and NF-kappa B signaling pathway (Fig. 3C, Supplementary Table S6).

### Identification of diagnostic marker genes for NAFLD

**Fig. 4:**
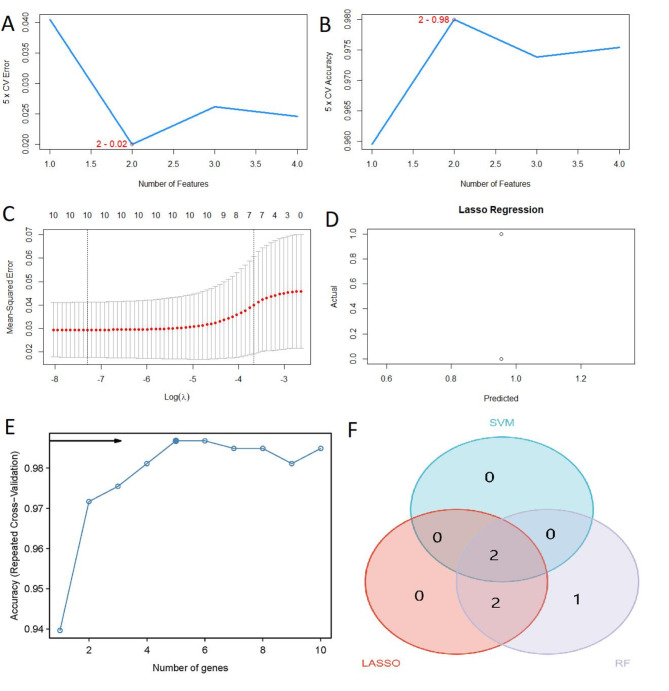
Machine learning identification of diagnostic marker genes for NAFLD. The accuracy and error rate of feature selection of the SVM algorithm reached the lowest cross-validation error of 0.02% (**A**) and the peak accuracy of 0.98% (**B**) when 2 genes were selected. (**C**) LASSO coefficient analysis. (**D**) Diagnostic performance of LASSO model. (**E**) Random forest analysis was used for 10 DE-PRGs, and 5 genes were included, with an accuracy of 0.99. (**F**) Venn diagram showing overlapping genes obtained using the three machine learning algorithms (SVM, LASSO, and RF). *SVM* support vector machine, *LASSO* least absolute shrinkage and selection operator, *RF* random forest, *NAFLD* nonalcoholic fatty liver disease.

Considering the individual complexity and heterogeneity of NAFLD patients and healthy controls, candidate key genes were identified from 10 DE-PRGs using LASSO regression and two validated machine learning models (SVM-RFE and RF), which aided in predicting NAFLD diagnosis. Two features were identified by SVM, (Fig. 4A and B). Four DE-PRGs were identified by the LASSO logistic regression algorithm, the coefficients of these four genes were non-0 in lasso regression model (Fig. 4C and D, Supplementary Table S7). And ten DE-PRGs were analyzed with RF, five of which were identified (Fig. 4E). A Venn diagram was used to intersect the essential genes in the LASSO, SVM-RFE, and RF analyses, identifying two key genes (TIRAP and GSDMD) for further analysis (Fig. 4F).

### Evaluation of the diagnostic performance of NAFLD Diagnostic marker genes

**Fig. 5:**
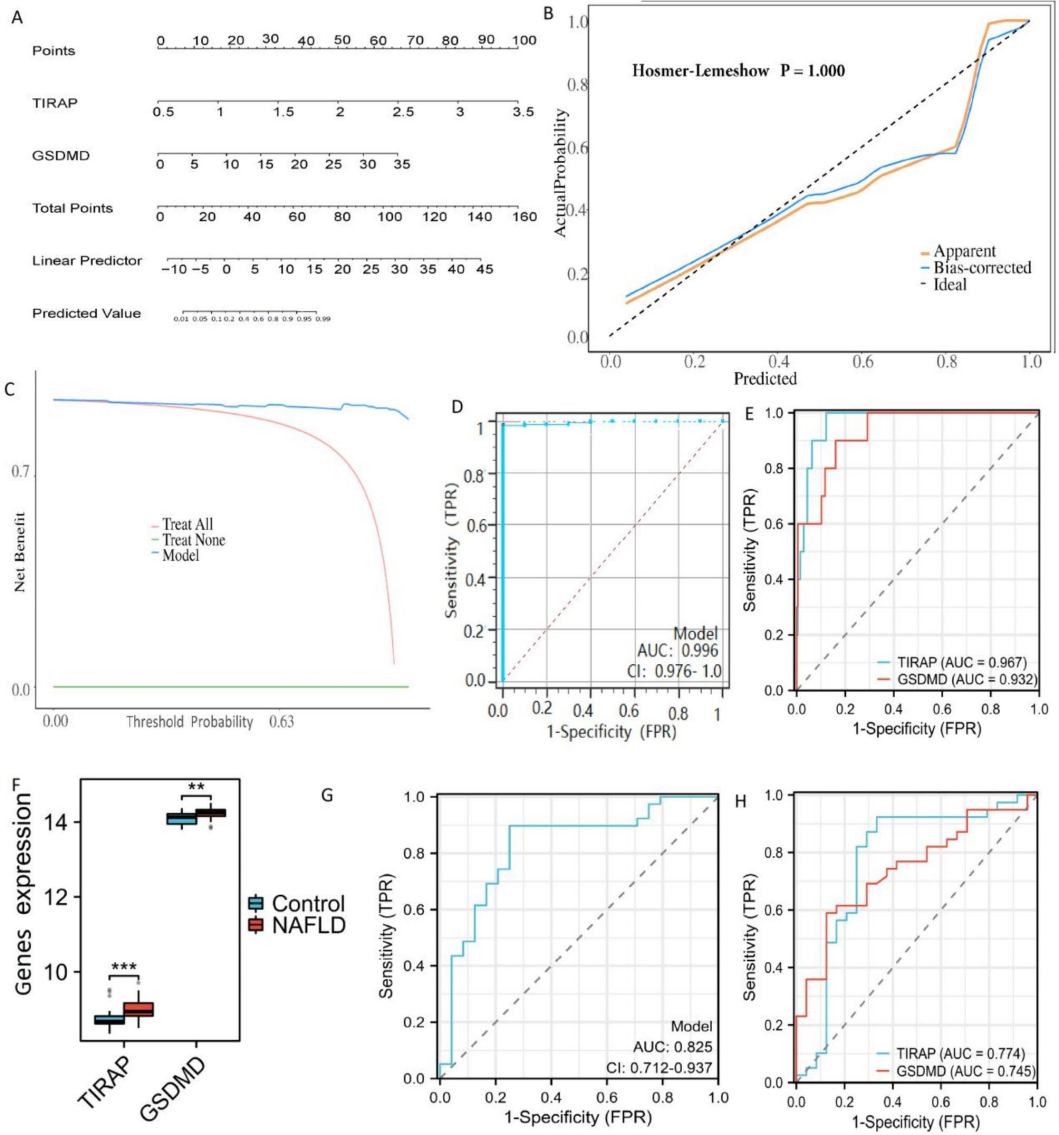
Establishment and verification of marker genes diagnostic model. (**A**) Nomogram of marker genes. (**B**) Calibration curve. (**C**) The predictive efficiency of the nomogram model was illustrated by DCA. (**D**) The ROC of the combination of the two key genes for the diagnosis of NAFLD was 0.996 (95% CI 0.976–1.0). (**E**) The ROC results of each of the two key genes for the diagnosis of NAFLD. The AUC values of TIRAP, and GSDMD were 0.967 and 0.932, respectively. (**F**) Boxplots of GSE89632 revealed that the two DE-PRGs between the NAFLD and control samples were significantly different. (**G**) The two-gene model had the ROC result with an AUC value of 0.825 in GSE89632. (**H**) The AUC value of TIRAP and GSDMD was 0.774 and 0.745, respectively, in GSE89632. Model: A model that combines two key genes (TIRAP and GSDMD) to diagnose NFALD. Note that “all” and “none” are the two reference strategies used to compare the benefits of the forecast model. The “all” reference strategy means treatment in all cases, while the “none” reference strategy means no treatment. The purpose of these two reference strategies is to help evaluate the benefit and clinical utility of the predictive model. *NAFLD* nonalcoholic fatty liver disease.

A nomogram model for the diagnosis of NAFLD was constructed, which included two central genes, TIRAP and GSDMD (Fig. 5A). The nomogram model’s numerical value for each biomarker was used to predict NAFLD risk, with a correction curve indicating a clear correlation between the predicted and actual probability (Fig. 5B). The DCA revealed that the net benefit from this model was significantly higher than 0, implying its remarkable accuracy and utility for clinical decision-making (Fig. 5C). The ROC curve analysis showed that the combined features of the two key genes demonstrated high performance in diagnosing NAFLD (AUC = 0.996, Fig. 5D) and the individual predictive ROC results for these two genes all exceeded 0.90 (Fig. 5E). The expression of 2 key genes in different groups of the GSE89632 dataset was shown in Fig. 5F. The ROC curve for the combination of the two genes in the GSE89632 sets was 0.825 (Fig. 5G), which was higher than the ROC curve for the predicted performance of the two genes separately (Fig. 5H). These indications suggest that the model based on these two marker genes may have strong predictive efficacy for NAFLD.

### GSEA and ssGSEA anlysis

**Fig. 6:**
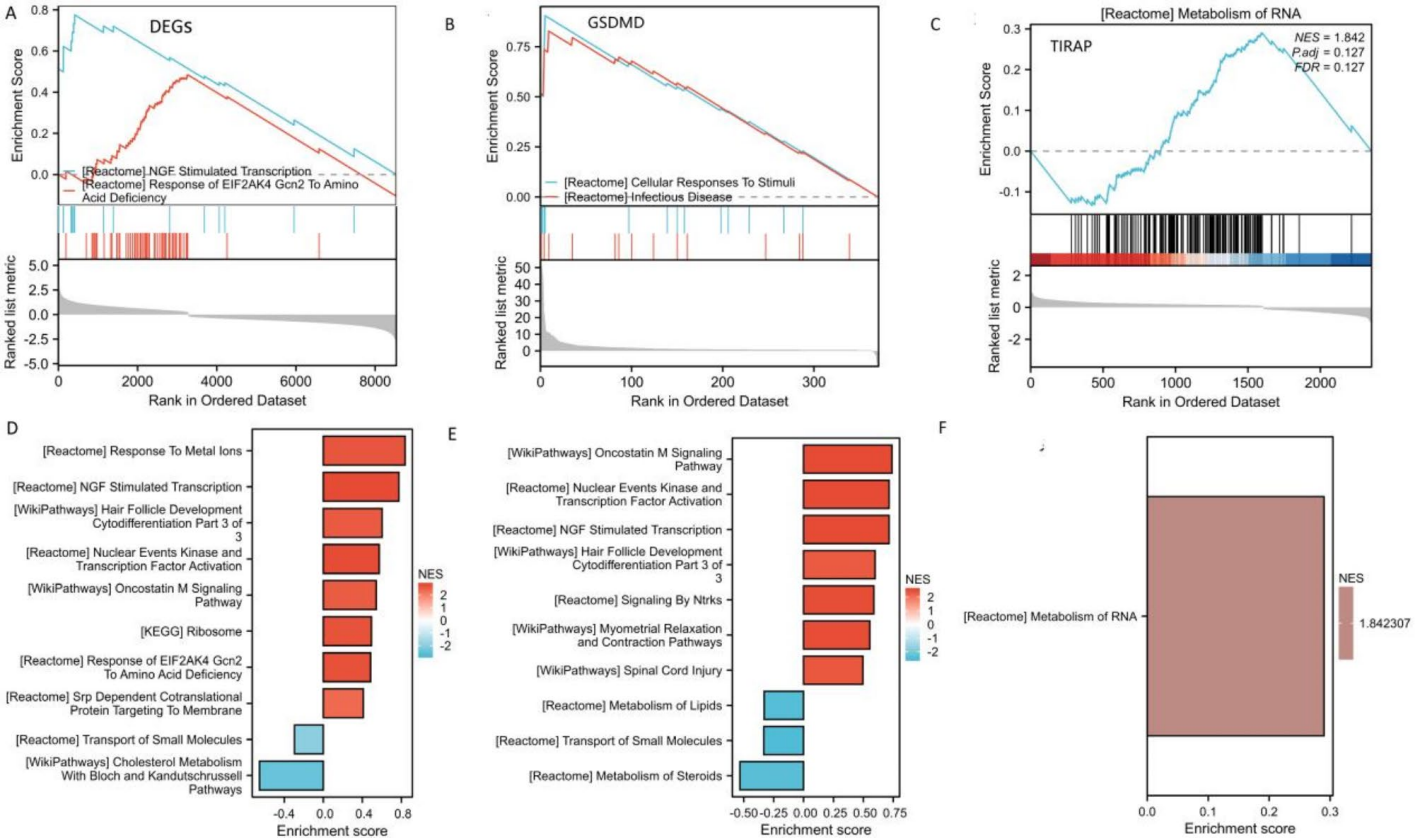
An enrichment analysis of the DEGs and the two marker genes. Classical graphs for GSEA analysis of the signature based on (**A**) DEGs, (**B**) GSDMD, and (**C**) TIRAP. Histogram for GSEA analysis of the signature based on (**D**) DEGs, (**E**) GSDMD, and (**F**) TIRAP. *GSEA* gene set enrichment analysis, *DEGs* differentially expressed genes.

We used GSEA to identify the major signaling pathways of the DEGs. GSEA of the KEGG pathways demonstrated that DEGs are implicated in NGF stimulated transcription, EIF2AK4 Gcn2 to amino acid deficiency, and metal ions (Fig. 6A and D, Supplementary Table S8). We used GSEA to identify the major signaling pathways of the two genes in the above model. GSEA of the KEGG pathways demonstrated that these two genes are implicated in cellular response to starvation. and infectious disase. GSVA revealed distinct activity pathways between low- and high-expression subtypes determined according to the levels of the two hub genes. Our analysis revealed that overexpression of GSDMD is involved in oncostatin M signaling, NGF stimulated transcription, and nuclear events kinase and transcription factor activation. Low GSDMD and TIRAP expression levels were linked to metabolism of lipids, small molecules metabolism of steroids, and metabolism of RNA (Fig. 6B–C E-F).

**Fig. 7:**
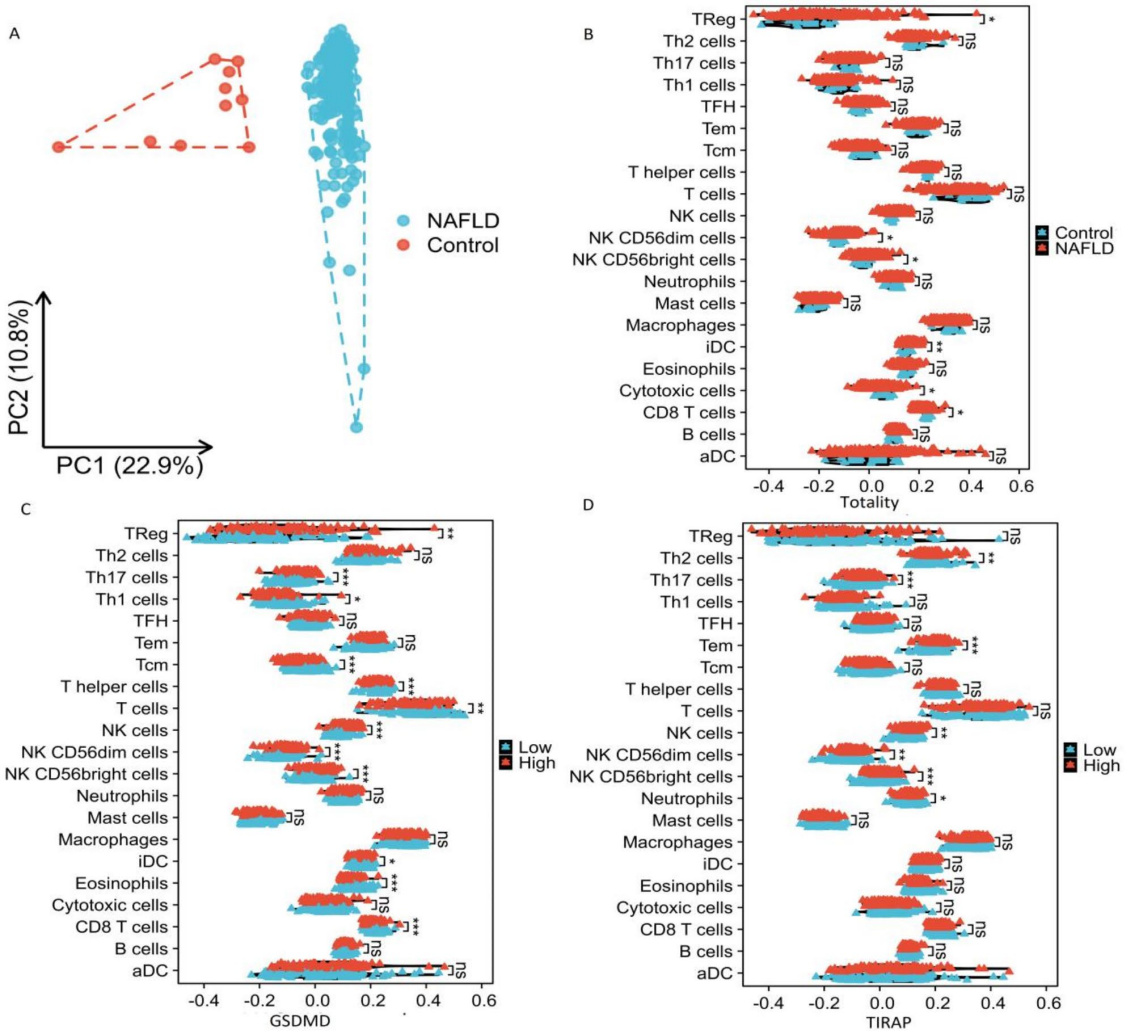
The expression of immune cells. (**A**) Principal component analysis further revealed a significant difference between NAFLD and control (206 NAFLD and 10 control samples in GSE135251). (**B**) Expression of different immune cells in NFALD and control. Expression of different immune cells in NAFLD with high and low expression of (**C**) GSDMD, (**D**) TIRAP. *NAFLD* nonalcoholic fatty liver disease.

In order to verify whether pyroptosis could promote NAFLD progression by mediating immune infiltration, we conducted ssGSEA analysis. According to the grouping of NAFLD and Control, the samples of 206 NAFLD and 10 Control were divided into two clusters (Fig. 7A). ssGSEA analysis showed that NK CD56 dim cells, iDC, Cytotoxic cells were significantly increased in NAFLD patients versus normal liver tissue (Fig. 7B). But, CD8 T cells and T-helper cells were the opposite. In addition, we investigated the relationship between immune cell infiltration and two DE-PRGs by ssGSEA. The two genes were divided into high expression group and low expression group, and many kinds of immune cells showed significant differential expression (Fig. 7C-D).

### Identification of drug candidates and ceRNA networks based on marker genes

**Fig. 8:**
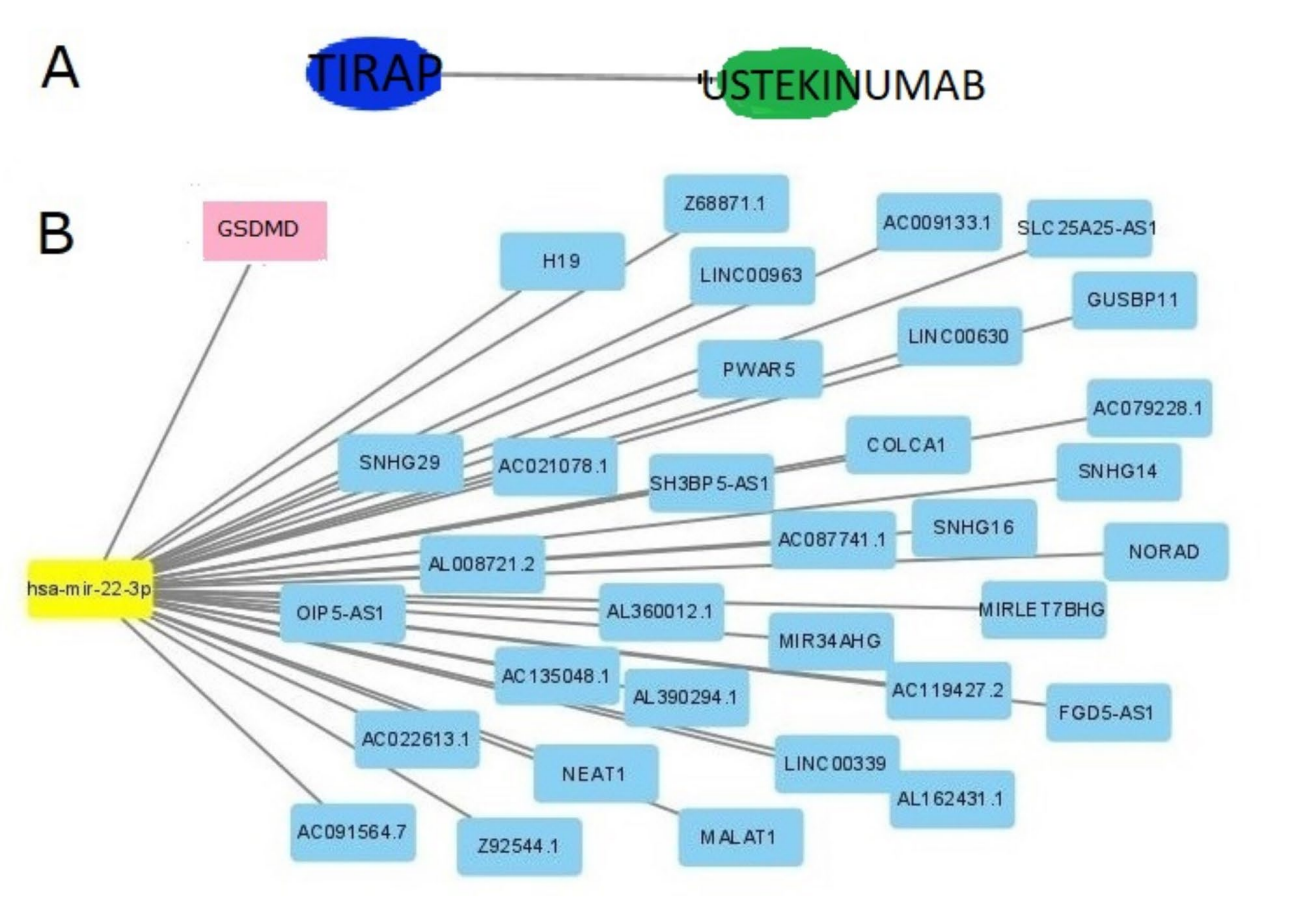
mRNA–Drugs and ceRNA Network. (**A**) The green rectangle nodes symbolize the drugs, while the mRNA–drug interaction network is represented by blue dots. (**B**) The ceRNA network, based on marker gene, is depicted with yellow dots for miRNA and baby blue dots for lncRNA.

To further explore drug therapy options for NAFLD, we analyzed the interactions between key genes and drugs using DGIdb. Cytoscape analysis revealed the interaction between genetic markers and drugs (Fig. 8A). A ceRNA network was constructed with the two essential genes using the TargetScan, miRanda, and miRDB databases, revealing one miRNA and 34 lncRNAs (Fig. 8B).

### Expression of PRGs in a cell model of NAFLD

**Fig. 9:**
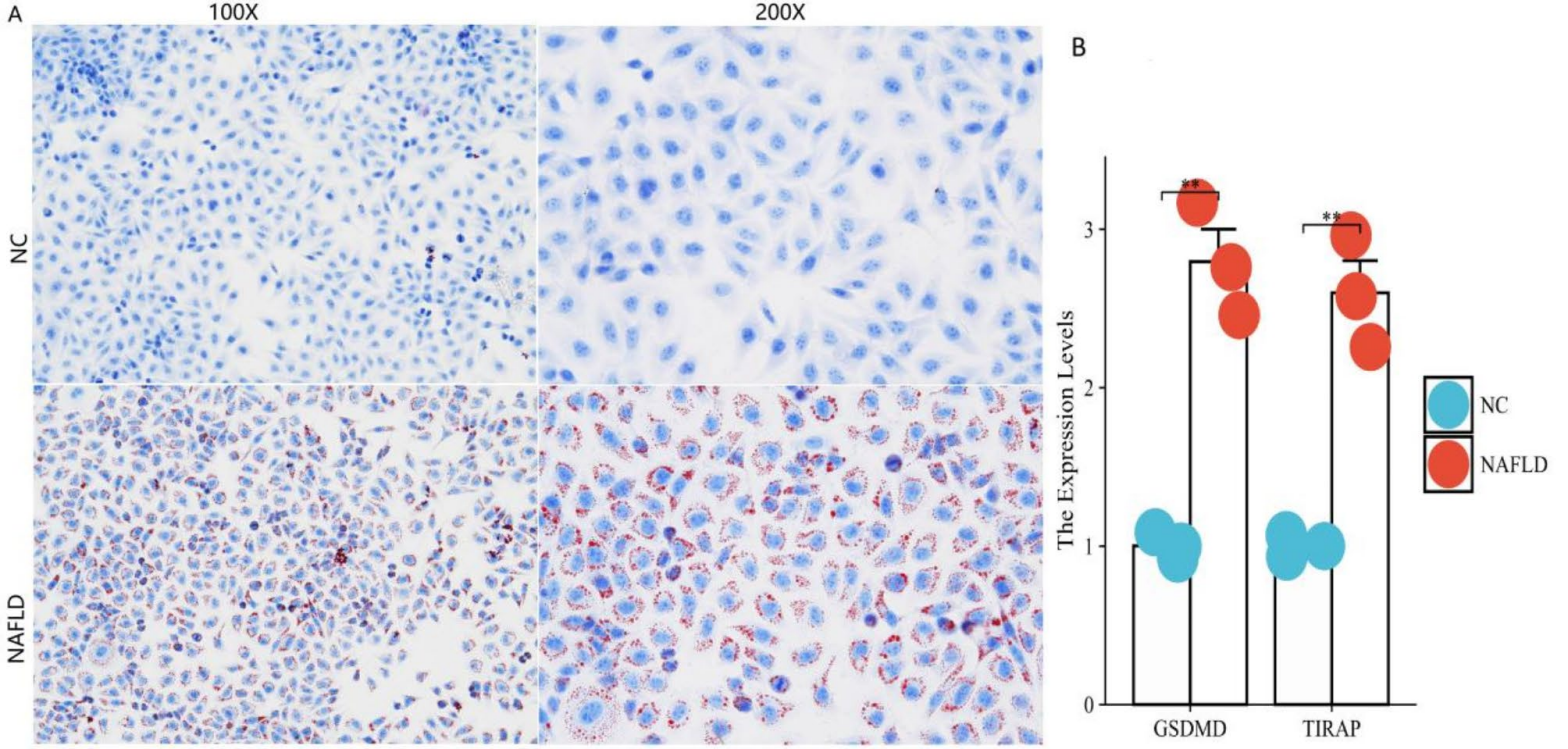
Expression of two key genes in Cell NAFLD Model. (**A**) Oil Red O staining. (**B**) The relative mRNA expression of the two hub genes in cell NAFLD model was verified by qRT‒PCR. *N* = 3, **p < 0.01.

Oil red O staining showed large lipid deposits in the NAFLD group cells, which were characterized by the formation of more fat droplets (Fig. 9A). qRT‒PCR measurement of mRNA levels indicated that the expression levels of the two key genes were significantly increased in the NAFLD group compared with those in the control group (Fig. 9B).

## Discussion

Non-alcoholic fatty liver disease (NAFLD) poses a significant global health challenge^32,33^. NAFLD poses a significant global health challenge^34–36^. However, the specific role of pyroptosis in the pathogenesis and regulation of NAFLD is still not fully understood. In this study, we investigated the potential role of PRGs in NAFLD, identified potential key genes, and explored possible target drugs.

We downloaded NAFLD and control liver data from the GEO database for statistical analysis to identify DEGs, resulting in the identification of 10 DEGs associated with pyroptosis levels. These findings suggest that PRGs may influence the progression of NAFLD. Our correlation analysis revealed that the identified DE-PRGs were closely related to each other; however, some showed no apparent correlation at the protein level, indicating heterogeneity in the interaction of PRGs at the gene and protein levels.

The important role of DE-PRGs inresponse to LPS,, cysteine-type endopeptidase activity, membrane raft, and Lipid and atherosclerosis was revealed by GO and KEGG enrichment analyses, respectively. LPS, also known as endotoxin, is a major component of the outer membrane of Gram-negative bacteria. LPS plays a critical role in the pathogenesis of various inflammatory diseases, including NAFLD^37^. In the context of NAFLD, this LPS-mediated inflammation is a key driver of liver damage. Recent studies have underscored the importance of the gut-liver axis in the progression of NAFLD^37^. Increased intestinal permeability allows for the translocation of LPS from the gut into the bloodstream, where it can reach the liver and exacerbate inflammation, contributing to the progression from simple steatosis to non-alcoholic steatohepatitis (NASH), a more severe form of NAFLD characterized by inflammation and fibrosis^38^.

Furthermore, interventions aimed at reducing LPS levels or blocking its signaling pathway have been shown to attenuate liver inflammation and fibrosis in NAFLD models^39^. The dysregulation of lipid metabolism, including increased de novo lipogenesis, impaired fatty acid oxidation, and altered lipid export, contributes significantly to hepatic fat accumulation^40^. an excess of saturated fatty acids can induce lipotoxicity, leading to hepatocyte injury, inflammation, and fibrosis^41^. Additionally, the role of cholesterol and its metabolites in NAFLD has been increasingly recognized. Cholesterol accumulation in the liver exacerbates hepatic inflammation and fibrosis, further contributing to NASH progression^42^.Cysteine-type endopeptidases, which belong to the family of proteases known as caspases, play a pivotal role in various cellular processes, including apoptosis, inflammation, and autophagy^43^. In the context of NAFLD, cysteine-type endopeptidase activity has been implicated in the progression of liver injury through mechanisms involving apoptosis and inflammation^44^.

Analyses using LASSO, RF, and SVM-RFE of the 10 DE-PRGs identified two key genes (TIRAP and GSDMD) that can effectively predict NAFLD, with an AUC value of 0.996. The validity of this two-gene model was confirmed using an external dataset, yielding AUCs of 0.825. The AUC values for the two key genes in the validation dataset exceeded 0.9. The nomogram model, calibration curves, and DCA demonstrated that this model possesses strong predictive capability and significant clinical applicability. Therefore, a predictive model incorporating these two key genes could serve as a reliable and robust biomarker for the effective prediction of NAFLD. TIRAP affects liver inflammation and immune response mainly by regulating Toll-like receptor (TLRs) signaling pathway^45^. In hepatitis, the expression of TIRAP is up-regulated, which may exacerbate the inflammatory response of the liver and lead to the aggravation of liver injury^46^. In addition, TIRAP is also involved in the development of liver fibrosis and promotes extracellular matrix accumulation by regulating the activation and proliferation of hepatic stellate cells^47^. GSDMD consists of an n-terminal domain (NTD, containing 242 amino acids) and a C-terminal domain (CTD, containing 43 amino acid splice and 199 amino acids)^48^. GSDMD (also known as GSDMDC1, DFNA5L, or FKSG10) was originally found in a congener of GSDMA^49^. Saeki et al. found that GSDMD is widely expressed in different tissues and immune cells^50,51^. The gasdermin protein family plays an important role in pyrodeath, and GSDMD is a key executive factor^52,53^. In NAFLD and NASH, GSDMD-mediated inflammatory cell death may exacerbate liver inflammation and liver injury^54^. In addition, GSDMD is also involved in the development of liver fibrosis by promoting the activation and proliferation of hepatic stellate cells and the accumulation of extracellular matrix^55^.

We conducted gene-targeting drug analysis based on the two key genes identified. A drug targeting the TIRAP gene was immunomodulatory drug. Given the interaction and influence of lncRNAs, miRNAs, and mRNAs on cellular biosynthesis^56–58^, we constructed an mRNA–miRNA–lncRNA regulatory network for NAFLD. This revealed that lncRNAs could regulate the one key gene (GSDMA). Therefore, gene-targeted drug analysis offers a novel approach to further search for potential drugs to prevent and treat NAFLD, and ceRNA network analysis provides a new pathway for further exploring the pathogenesis of NAFLD. These findings, however, require further validation in cell and animal studies.

Our study does have some limitations. Firstly, we performed genetic analysis on data downloaded from the GEO database, which may contain certain biases. Secondly, the total number of cases was relatively small. Furthermore, we have not yet performed cellular or animal validation of the gene-targeting drugs we discovered.

## Conclusions

We initially identified four significant genes, and by combining these two genes, we can accurately diagnose patients with NAFLD. We then explored the relationship between these genes and invasive immune cells and analyzed the significant heterogeneity in immune responses between NAFLD patient and control liver samples. Our research unveils the role of pyroptosis in NAFLD, providing a new theoretical foundation for the potential pathogenesis of NAFLD and therapeutic options.

## Electronic supplementary material

Below is the link to the electronic supplementary material.

Supplementary Material 1

Supplementary Material 2

Supplementary Material 3

Supplementary Material 4

Supplementary Material 5

Supplementary Material 6

Supplementary Material 7

Supplementary Material 8

## Data Availability

“The datasets in this study were enrolled from the GEO database (https://www.ncbi.nlm.nih.gov/geo/), with the following data accessions enrolled: GSE135251 and GSE89632. The datasets used and/or analysed during the current study are available from the corresponding author on reasonable request.”

## Abbreviations

ALT: Alanine aminotransferase
AST: Aspartate aminotransferase
AUC: Area under curve
BP: Biological processes
CC: Cellular component
PRG: Pyroptosis-related gene
DCA: Decision curve analysis
DE-PRG: Differential expression of pyroptosis-related gene
DEG: Differential expression genes
DGIdb: Drug–gene interaction databases
DSigDB: Drug signatures database
GEO: Gene expression omnibus
GO: Gene ontology
GSEA: Gene set enrichment analysis
GSVA: Gene set variation analysis
KEGG: Kyoto Encyclopedia of Genes and Genomes
LASSO: Least absolute shrinkage and selection operator
MF: Molecular functions
NAFLD: Non-alcoholic fatty liver disease
PPI: Protein‒protein interaction
RF: Random forest
ROC: Receiver operating characteristics
ssGSEA: Single-sample gene set enrichment analysis
SVM-RFE: Support vector machine-recursive feature elimination
