# Supplementary material for: Identification of diagnostic markers pyrodeath-related genes in non-alcoholic fatty liver disease based on machine learning and experiment validation
Source: Sci Rep. 2024 Oct 26;14:25541. doi: 10.1038/s41598-024-77409-3 (PMC11513955; doi:10.1038/s41598-024-77409-3)
Supplement: Supplementary file 1 — Supplementary Material 1 [file 41598_2024_77409_MOESM1_ESM.docx]

Table S1 The primers used for cell

| **Gene** | **Primer** | **Sequence (5'-3')** | **PCR Products** |
| --- | --- | --- | --- |
| Homo b-actin | Forward | CCCTGGAGAAGAGCTACGAG | 180bp |
|  | Reverse | CGTACAGGTCTTTGCGGATG |  |
|  | Reverse | TTGGGTTTTCCAGTTAGAC |  |
| Homo GSDMD | Forward | GGAGCTTCCACTTCTACGATG | 165bp |
|  | Reverse | GAGTCTGCCAGGTGTTAGGG |  |
|  | Reverse | AAGGTCCTCTGCTTCTTATCC |  |
| Homo TIRAP | Forward | ACTCCCCAGAAAGCACCTC | 292bp |
|  | Reverse | TGGCACAGCTCGGACACTA |  |
